# Embodied Energy Use in China's Infrastructure Investment from 1992 to 2007: Calculation and Policy Implications

**DOI:** 10.1100/2012/858103

**Published:** 2012-12-20

**Authors:** Hongtao Liu, Youmin Xi, Bingqun Ren, Heng Zhou

**Affiliations:** ^1^School of Management, Northwestern Polytechnical University, Xi'an 710072, China; ^2^School of Management, Xi'an Jiaotong University, 28 West Xianning Road, Beijing District, Xi'an 710049, China; ^3^School of Economics and Finance, Xi'an Jiaotong University, Xi'an 710061, China

## Abstract

Infrastructure has become an important topic in a variety of areas of the policy debate, including energy saving and climate change. In this paper, we use an energy input-output model to evaluate the amounts of China's embodied energy use in infrastructure investment from 1992 to 2007. We also use the structure decomposition model to analyze the factors impacting the embodied energy use in infrastructure investment for the same time period. The results show that embodied energy use in infrastructure investment accounted for a significant proportion of China's total energy use with an increasing trend and reflect that improper infrastructure investment represents inefficient use of energy and other resources. Some quantitative information is provided for further determining the low carbon development potentials of China's economy.

## 1. Introduction

**Figure 1:**
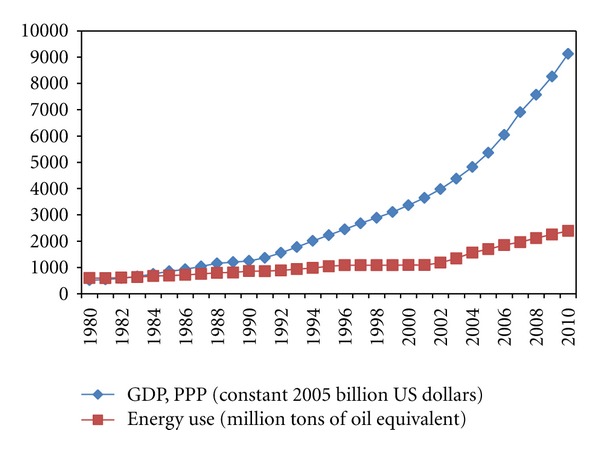
China's energy use and GDP from 1980 to 2010.

During the last three decades, China's remarkable economic growth not only has enabled it to achieve social progress, but also has been accompanied by a corresponding surge in energy use (Figure 1). Although China has successfully declined its energy intensity (energy consumption per unit gross domestic product) by 67% from 1980 to 2010, it is now the world's largest energy consumer and biggest emitter of carbon dioxide (CO_2_), the chief greenhouse gas (GHG) [1]. Hence, China is facing immense energy related pressures and challenges, such as energy supply shortage, high foreign dependency for oil, massive acid deposition, and growing international pressure about GHG emissions reduction [2, 3].

The adequate supply of infrastructure services has long been recognized as an essential ingredient for productivity improvement and economic growth [4, 5]. For China, there is persuasive evidence that sufficient infrastructure provision is a key element to achieve its intended objective of export growth [6]. Also, increasing access to infrastructure services in China has played a key role in helping reduce income inequality and increase efficient resource reallocation [7]. China has undergone a remarkable economic growth with an annual growth rate over 10% from 1980 to 2010, which is mainly driven by sustained increase in domestic investment and a massive development of physical infrastructure [8, 9]. However, infrastructure investment will not only bring a large amount of energy consumption directly and will also result in energy consumption indirectly through the use of cement, iron, steel, and other energy-intensive products. The role of infrastructure investment played on energy use has received increased attentions.

Either the input-output model or life-cycle assessment model could be established to quantitatively evaluate the impacts of infrastructure construction on energy use. But the application of Life-Cycle Assessment has been limited by data availability in practice [2, 3, 10]. Input-output analysis is a useful analytical framework developed by Leontief [11]. It uses input-output table to estimate the direct and indirect impacts of one economic sector's output changes on other sectors [12–14]. Therefore it can conveniently evaluate the quantitative relationships among all economic sectors, including the energy producers and its users [15–17].

In recent years, input-output analysis has been widely applied in evaluating the energy consumption caused by different economic activities in national or regional economies [18, 19]. Pick and Becker [20] applied input-output analysis to evaluate direct and indirect uses of energy and materials in engineering and construction. Nässén et al. [21] use top-down input-output analysis to assess direct and indirect energy use as well as carbon emissions in the Swedish building sector and compared the results to that from 18 previous bottom-up studies using process-LCA methodology. For China's case, many scholars have already studied the impacts of different economic activities on energy consumption. Polenske and McMichael [22] use input-output analysis to analyse the energy consumption and environmental pollution in China's coke-making industry. Liu et al. [16] comprehensively evaluated households' indirect energy consumption and impacts of alternative energy policies in China. Liange et al. [23] propose a hybrid physical input-output model to study energy metabolism by taking Suzhou in China as an example.

However, few analysts have studied the infrastructure investment impacts on energy consumption. In this paper, we measure the energy use embodied in China's infrastructure investment, which aims to provide critical insights for the country's policy-makers to refine the current intensity-reduction-oriented energy-efficiency policies. We first build an energy input-output model to identify quantitatively the amounts of China's energy use embodied in infrastructure in 1992, 1997, 2002, and 2007. We also use the model to analyze the key factors driving the growth of energy use embodied in infrastructure for the same period.

## 2. Energy Input-Output Analysis

Infrastructure could be defined as the basic physical systems needed for one country or one region's economy to function, including transportation, water, sewage, communication, and electric systems. According to national economic accounting, infrastructure investment is a part of GDP measured from the expenditure side [24]. Infrastructure investment plays an important role in expanding China's economic growth by providing increasing production conditions of various economic sectors. Like other economic activities, infrastructure investment consumes both energy and nonenergy goods and services, so that the energy consumed by infrastructure investment should take the embodied energy of all these goods and services into account.

### 2.1. Energy Input-Output Model

Beginning from the basic Leontief Input-output model, the total output of an economy, **X**, can be expressed as the sum of intermediate consumption, **A** **X**, and final consumption, **Y** [11]:

(1) $$\begin{array}{c} X=AX+Y,\\ {\left(I-A\right)}^{-1}=B, \end{array}$$

where **X** is the *n* × 1 total output vector, **A** is the *n* × *n* direct input coefficient matrix, describing the interindustry relationships between all sectors of the economy, **Y** is the *n* × 1 final demand vector, and **B** is the Leontief inverse matrix, (**I** − **A**)^−1^. **A** **X** denotes the intermediate input vector, which can be obtained by multiplying the direct input coefficient matrix by the total output vector. The final demand vector, **Y**, can be treated as exogenous to the system; for example, the level of total production can be determined by the final demand (2):

(2) $$\begin{array}{c} Y=BX. \end{array}$$

Input-output model can be applied to calculate each sector's indirect energy consumption regardless of the length and complexity of their production processes by using the energy input-output table (Wu and Chen, 1990; Peet, 1993). In energy input-output tables, energy sectors should be represented both in monetary and energy terms for computing the direct energy consumption coefficient matrix [14]. Assume that in input-output tables the economy can be categorized into *n* sectors, which includes *k* energy sectors and *n*-*k* nonenergy sectors. Hence we can write an equation representing the way in which energy sectors distribute their products to energy sectors, nonenergy sectors, and final demands in physic units:

(3) $$\begin{array}{c} A_{k,1}+A_{k,2}+\cdots +A_{k,k}+A_{k,k+1}+\cdots A_{k,n}+f_{k}=x_{k}. \end{array}$$

Using energy input-output tables, the direct energy intensity and total energy intensity of each economic sector can be calculated. Direct energy intensity of one sector is calculated as the ratio of direct energy consumption (in physical terms) to total inputs (in monetary terms). Total energy intensities are calculated by multiplying direct energy intensity matrix with the Leontief inverse matrix of the corresponding energy input-output table. Embodied energy use in infrastructure investment can be calculated by multiplying total energy intensities with infrastructure investment:

(4) $$\begin{array}{c} e_{i}=\frac{\sum_{i=1}^{k}E_{j,1}}{X_{i}}, \end{array}$$

(5) $$\begin{array}{c} e_{}^{total}=e{\left(I-A\right)}^{-1}, \end{array}$$

(6) $$\begin{array}{c} E_{}^{II}=e_{}^{total}Y^{II}=e{\left(I-A\right)}^{-1}Y^{II}. \end{array}$$

*e*_*i*_ is the direct energy intensity of sector *i*, **e** is the direct energy intensity matrix, and **e** ^**total**^ is the total energy intensity matrix. **E** ^**II**^ is the embodied energy in infrastructure investment, and **Y** ^**II**^ is the infrastructure investment.

### 2.2. Structural Decomposition Analysis

Based on (6), the change of embodied energy use in infrastructure investment is driven by several factors, such as growth in infrastructure investment, energy efficiency improvement, and industrial structure changes. Aiming at identifying the driving factors for changes in embodied energy use in infrastructure investment overtime, we applied input-output structural decomposition analysis on (6). Beginning from the basic Leontief model, change in the embodied energy use in infrastructure investment can be expressed as follows:

(7) $$\begin{array}{c} \Delta E_{}^{II}=e_{t}{\left(I-A_{t}\right)}^{-1}Y_{t}^{II}-e_{t-1}{\left(I-A_{t-1}\right)}^{-1}Y_{t-1}^{II}, \end{array}$$

where Δ*E* ^*II*^ = *E*_*t*_^*II*^ − *E*_*t*−1_^*II*^, and Δ*E* ^*II*^ is the change of embodied energy use in infrastructure investment during the period [*t* − 1, *t*].

Equation (7) can be decomposed to analyze changes in embodied energy use in infrastructure investment over time. We use a common decomposition method to separate factors related ((8a), which represents aggregated changes in the direct energy intensities), the Leontief effect ((8b), which is change in intersector relationships), and infrastructure investment ((8c) which represents changes in infrastructure investment) [25–27].

(8a) Equation  (7)=et(I−At−1)−1Yt−1II−et−1(I−At−1)−1Yt−1II

(8b)  +et−1(I−At)−1Yt−1II−et−1(I−At−1)−1Yt−1II

(8c)  +et−1(I−At−1)−1YtII−et−1(I−At−1)−1Yt−1II

(8d)  +ε.

### 2.3. Data Input

This paper aims to analyse the embodied energy use in physical infrastructure investment, including investment in transport services, communication, energy supply, and water management. In order to carry out a detailed analysis of the impact of physical infrastructure investment, there is a need to disaggregate investment by sector. The datasets that will be used in our study are input-output tables, the Income and Expenditure Survey, and Investment Survey from the National Bureau of Statistics of China.

Given the energy input-output model, we constructed hybrid unit energy input-output tables [14] based on monetary input-output tables published by the National Bureau of Statistics of China from 1992 to 2007. In hybrid unit energy input-output tables, the energy sectors' products are presented both in physical units (e.g., tonnes of coal equivalent) and monetary terms, and the nonenergy sectors' products are presented only in monetary terms. The data of energy sectors' products are extracted from Chinese Energy Statistical Year books. To calibrate the data of the input-output tables and energy statistics, we adjust the sector classification of the input-output tables. For more details about data calibration of hybrid unit energy input-output tables, refer to [28].

## 3. Results and Discussion

### 3.1. Results

**Figure 2:**
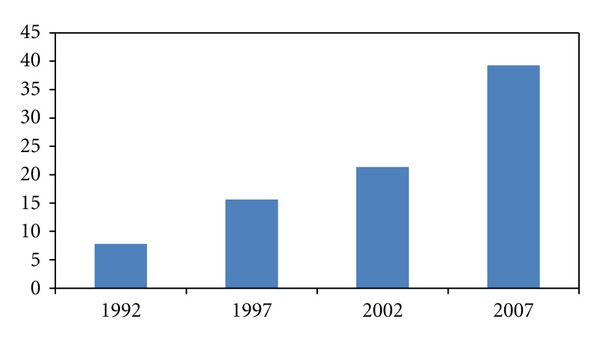
China's embodied energy use in infrastructure investment in absolute term (million tons of standard coal).

**Figure 3:**
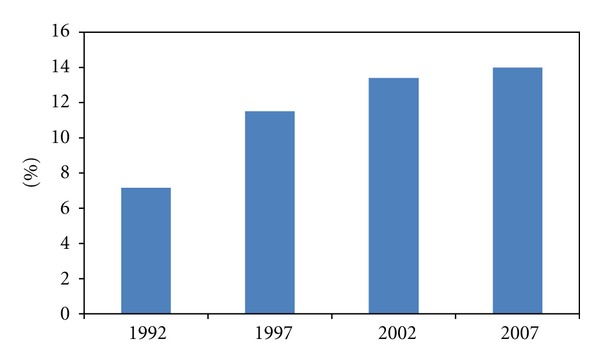
China's embodied energy use in infrastructure investment in percentage of its total energy use.

The overall results of China's embodied energy use in infrastructure investment are reported in Figures 2 and 3, in absolute term and percentage of China's total energy use, respectively. China's fast-increasing infrastructure investment, with annual growth rate 25% from 1992 to 2007, has led to accelerated requirements of energy. As shown in Figure 2, China's embodied energy use in infrastructure investment increased from 78 million tons of standard coal in 1992 to 354 tons of standard coal in 2007. The results also show that the embodied energy use in infrastructure investment has increased rapidly from 2002 to 2007. The embodied energy use in infrastructure investment growth from 2002 to 2007 was about 140 million tons of standard coal, which is more than the growth of embodied energy use in infrastructure investment from 1992 to 2002.

In terms of proportion, the embodied energy use in infrastructure investment accounted for 7.16% of China's total energy use in 1992, and the proportion increased to 13.4% in 2002. Then it increased to 14.0% in 2007, which means that it has accounted for a significant proportion of China's total energy use during our observation period and it is one of the key factors driving China's energy consumption growth. From 1992 to 2007, China's total energy use increased from 1.10 billion tons of standard coal to 2.81 billion tons of standard coal. Hence, the infrastructure investment could play an important role in inducing China's energy consumption as well as GHG emissions.

The structural decomposition results of China's embodied energy use in infrastructure investment are shown in Table 1. Energy efficiency improvement in China, indicated as decreasing in energy intensities, is the main factor to hinder the growth of embodied energy use in infrastructure investment. Changes of industrial structure also have decreased China's embodied energy use in infrastructure investment while the growth of China's infrastructure investment, which was the most significant impact factor, led to the increase of embodied energy use in infrastructure investment. Infrastructure investment activities, mainly occurred in the construction sector, usually consume a huge amount of energy-intensive materials, such as cement and steel, which causes significant indirect energy consumption from a life-cycle perspective. Therefore, these results indicate that in order to reduce the embodied energy use in infrastructure investment, it is important to decrease the energy use embodied in these energy-intensive materials, prolong the lifespan of infrastructure, and improve the design of infrastructure investment policies.

### 3.2. Discussion

Generally, infrastructure investment is considered to have significant positive multiplier (generative) effects on national economy, because it could not only improve the productivity, but also trigger investment from other economic sectors and ultimately increase national income. In recent years, for railway projects only, more than 4 trillion Yuan ($597 billion, 1 US dollar = 6.6 Yuan as in 2010) has been approved in China, and a large proportion of which targeted the high-speed rail lines (Ministry of Railways 2010). Except for the economic benefits associated with high-speed rail investment, high-speed rail is also considered as energy efficient and environment friendly since it is electrified and does not generate carbon emissions during operation. 

However, the claimed benefits associated with infrastructure investment, which are related to economic development or climate change mitigation goals, still need close inspection as well as quantitative research efforts. Take high-speed rail as an example; even though the direct energy use of high-speed rail is clean during the operation period, its direct energy consumption during the construction period is enormous, regarding the production and shipment of major building materials, that is, cement and steel. Moreover, it is true that high-speed rail is mostly electrified, but the supply of power is not necessarily low-carbon. In the case of China, because over 80% of the electricity is currently generated from coal, it is highly possible that, the reduction of energy intensity and carbon emissions along the high-speed rail corridor is at the cost of intensified energy use in regions where power plants locate. Finally, if in fact there are not enough passengers traveling on the high-speed rail line, the per capita energy consumption and carbon emissions could rise rapidly. 

Infrastructure projects incur huge amounts of upfront costs, but the environmental influences go beyond the project life cycle. The standard cost-benefit analysis framework used by China's development authorities could not captured these influences. A comprehensive and systematic assessment of energy use impacts of infrastructure investment is essential to understand the role of infrastructure investment in achieving the goals of climate change mitigation. For China, the central government should be aware of the temporary nature of the stimulus effects of infrastructure investment, even though the short-run impacts may be significant in magnitude. Because in the long run marginal returns to infrastructure improvement are decreasing and the direct and indirect energy use of infrastructure is huge. The government needs to seek for more sustainable driving forces of economic development and needs to be aware of the risk of overbuilding.

## 4. Conclusion

This paper aims at improve the understanding of the implications of China's infrastructure investment on its energy use. Based on the energy input-output analysis, we calculated the embodied energy use in infrastructure investment from 1992 to 2007. We also quantitatively analyzed the factors impacting the embodied energy use in infrastructure investment using a structure decomposition analysis. The results obtained from the energy input-output analysis show that an increasing trend of both China's energy embodied in infrastructure investment as well as the ratio of China's energy embodied in infrastructure investment to its total energy consumption during our observation period. The decomposition results show that energy efficiency improvement is the main reason for hindering the growth of energy embodied in infrastructure investment and the increase of infrastructure investment is the most important factor driving the growth of energy embodied in infrastructure investment.

Our results reflect that the infrastructure growth required by China's rapid urbanization and industrialization has consumed a large amount of energy. Given this fact, we can conclude that the problems of repetitive layout, improper location, mutual contradiction, and bad structure existing in China's infrastructure represent inefficient use of capital, energy, and other resources consumed by corresponding infrastructure investment activities. Chinese policy makers should improve their design of the country's infrastructure investment policies in terms of further determining the energy-saving potentials of China's economy from the perspective which we presented through this study.

## Figures and Tables

**Table 1:** The structural decomposition results of China's embodied energy use in infrastructure investment.

	1992–1997	1997–2002	2002–2007
Δ*I*_*ei*_	−64.90	−34.85	−58.01
Δ*I*_*is*_	−6.26	−1.20	−2.33
Δ*I*_*ii*_	149.35	93.17	239.40
Δ*I*_*eII*_	78.19	57.13	179.06

(a) Negative values indicate effects of decreasing embodied energy use in infrastructure investment.

(b) Δ*I*_*ei*_, Δ*I*_*is*_, Δ*I*_*ii*_ and Δ*I*_*eII*_ are the effect of direct energy intensities, the industrial structure effect, changes in infrastructure investment and change in embodied energy use in infrastructure investment respectively.
